# Rare Clinical Entity: Metastatic malignant struma ovarii diagnosed during pregnancy – Lessons for management

**DOI:** 10.1186/s40842-018-0064-5

**Published:** 2018-06-19

**Authors:** Corey J. Lager, Ronald J. Koenig, Richard W. Lieberman, Anca M. Avram

**Affiliations:** 1 Division of Metabolism, Endocrinology, and Diabetes, University of Michigan, 24 Frank Lloyd Drive, SPC 451. Domino’s Farms Lobby C, Suite 1300, Ann Arbor, MI 48106 USA; 2 Division of Metabolism, Endocrinology, and Diabetes, University of Michigan, Cancer Center Floor 1, 1500 E Medical Center Dr SPC 5354, Ann Arbor, MI 48109 USA; 3 Department of Obstetrics and Gynecology and Department of Anatomic Pathology, University of Michigan, Von Voigtlander Hospital Floor 9 Clinic B, 1540 E Hospital Dr SPC 4276, Ann Arbor, MI 48109 USA; 4 Department of Nuclear Medicine, University of Michigan, 1500 E Medical Center Dr, B1G505, Ann Arbor, MI 48109-5028 USA

**Keywords:** Struma ovarii, Thyroid cancer, Radioactive iodine, Thyroid imaging, Pregnancy

## Abstract

**Background:**

Malignant struma ovarii is an ovarian teratoma containing at least 50% thyroid tissue which has the potential to metastasize and produce thyroid hormone. Given its rarity, management strategies are not well-established. We report a case of metastatic malignant struma ovarii discovered during pregnancy with lessons for evaluation and management.

**Case presentation:**

A 30-year-old woman who was two months pregnant was discovered to have struma ovarii with over half of the struma comprised of papillary thyroid cancer. Following tumor resection, delivery, and thyroidectomy, she underwent evaluation with stimulated thyroglobulin testing and diagnostic staging sodium iodide-131 scan (I-131), which revealed the presence of skeletal metastases. Following administration of 320 mCi I-131, post-therapy scan also showed miliary pulmonary metastases with improved ability to localize the bony and pulmonary metastases with concurrent SPECT/CT imaging. A second dosimetry-guided I-131 therapy resulted in complete resolution of pulmonary metastases; however, small foci of residual bone disease persisted. Post-therapy scans demonstrated additional findings not shown on diagnostic I-131 scans obtained prior to both her initial and second I-131 therapy.

**Conclusions:**

SPECT/CT provides accurate anatomic correlation and localization of metastatic foci and can serve as a baseline study to assess interval response to treatment. Post-therapy scans should always be obtained when I-131 treatment is administered, as additional findings may be revealed versus low dose I-131 activity diagnostic scans. This patient had a high metastatic burden that would not have been discovered in a timely fashion with the conservative approach advocated by others. Thyroidectomy followed by a diagnostic staging radioiodine scan and a stimulated thyroglobulin level should be considered in patients with malignant struma ovarii for guiding therapeutic I-131 administration as metastatic risk is difficult to predict based on histopathologic examination.

## Background

Struma ovarii is a rare form of ovarian teratoma (ovarian dermoid cyst) that contains greater than 50% thyroid tissue, accounting for less than 1% all ovarian tumors [1]. Hyperthyroidism is present in 5–8% of cases, while thyroid cancer is uncommon, representing less than 5% of all struma [1, 2] with distant metastases being reported in 5–23% of patients with malignant struma ovarii [3]. The pathologic diagnosis of malignant transformation within ovarian dermoid cysts is challenging as the histologic criteria for cancer within the thyroid gland may not be predictive of metastatic potential in struma ovarii [4]. The diagnostic criteria and management strategies are controversial owing to the rarity of this condition and lack of reliable predictive factors regarding metastatic potential. We present the case of a patient diagnosed with malignant struma ovarii during pregnancy and how a more aggressive approach was beneficial. We highlight the benefits of diagnostic radioiodine scintigraphy with single photon emission computed tomography/computed tomography (SPECT/CT) for completion of staging and for guiding sodium iodide-131 (I-131) treatment based on dosimetry calculations. We also discuss the importance of post-therapy I-131 scans for assessing disease burden and present the follow-up strategy used for ascertaining therapeutic response. The role of estrogen, progesterone, and human chorionic gonadotropin (hCG) stimulation in thyroid cancer is also reviewed.

## Case presentation

**Fig. 1:**
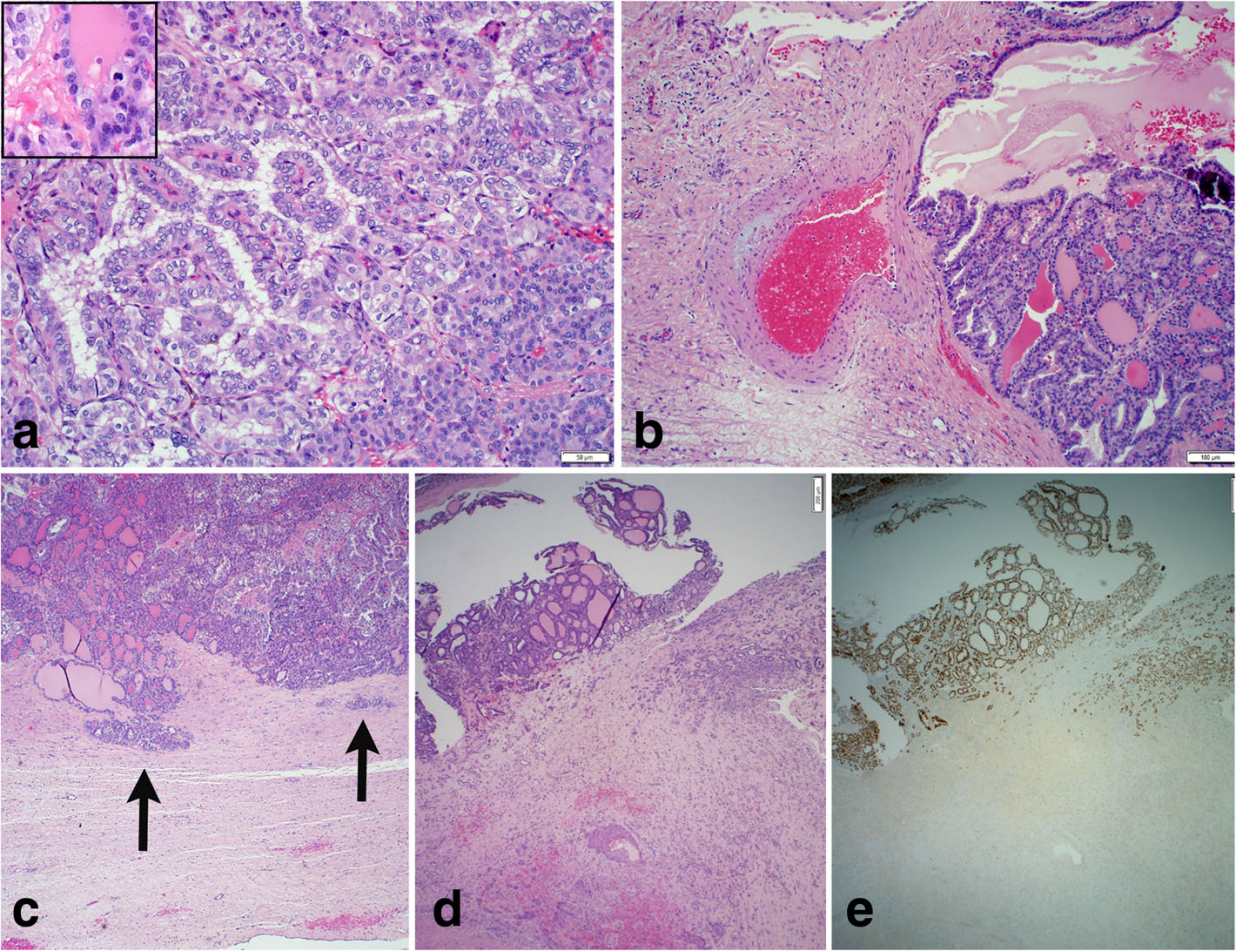
Histologic sections of malignant struma ovarii. Areas of adenomatous differentiation (**a** - lower right) and classic papillary thyroid carcinoma (**a** - center left) with papillary growth and cleared out “orphan Annie” nuclei. Area of Hurthle cell differentiation (**a** – center right) with an illustrative mitosis (**a** – inset). Tumor proliferation impinging on vascular structures (**b**). While the irregular border of the tumor and myxoid degeneration of the vessel wall are suggestive of infiltrative growth, no definitive vascular invasion is identified in the histologic sections reviewed. Tumor proliferation extends into the ovarian capsule (arrows) without extra-capsular extension (**c**). Struma ovarii (**d** - upper left) and squamous differentiation of the mature cystic teratoma (**d** - mid-lower right). Immunohistochemical stain for thyroid transcription factor (TTF-1) at 40× magnification with positive staining only in the areas of thyroid differentiation (**e**). **a**-**d** are hematoxylin and eosin stained with magnification 200× (**a**), 100× (**b**), and 40× (**c**, **d**)

A 30-year old woman presented with two weeks of left lower quadrant discomfort during her second month of pregnancy. Ultrasound revealed a mixed cystic and solid left adnexal mass measuring 8.6 × 6.7 × 8.3 cm. Left salpingo-oophorectomy was performed early in the second trimester. Grossly, the left ovary demonstrated an intact 7.5 cm complex cystic-solid mass, with nearly half involved by a friable, tan-yellow tumor with papillary excrescences, some of which were freely floating within the cyst cavity. Histologic sections demonstrated a struma ovarii with over 50% characterized by the classic features of a well-differentiated papillary thyroid carcinoma (Fig. 1A). The periphery of the tumor impinged on the ovarian capsule and vascular structures; however, no definitive evidence of invasion was seen on the histologic sections reviewed (Fig. 1B-C). Typical histology for an ovarian mature cystic teratoma (such as areas of squamous differentiation) were present in other areas of the tumor (Fig. 1D). The tumor expressed positive immunohistochemical staining for thyroglobulin (Tg) and thyroid transcription factor 1 (TTF1, Fig. 1E), but was negative for BRAF V600E. Serum Tg was 83.5 ng/mL (reference range for euthyroid status in nonpregnant adults 0–35 ng/mL) seven days after surgical resection of the ovarian mass. The elevated Tg level is reasonably expected given the proximity to surgery and Tg half-life of 65 h [5] and the known increase in Tg levels during pregnancy [6, 7]. Thyroid function tests (TSH 1.66 mU/L) and thyroid ultrasound were normal. Levothyroxine (L-T4) was started to suppress thyroid stimulating hormone (TSH) to 0.1–0.5 mU/L although TSH ranged 0.86–1.41 mU/L during pregnancy. After delivery, Tg was 2.9 ng/mL (TSH 0.57 mU/L). With thyroglobulin levels in a reasonable range as above, the risk of additional studies during pregnancy in terms of radiation or hypothyroid exposure, unknown risk of metastatic disease, and unknown incremental benefit of intervention during pregnancy, the decision was made to delay further testing and therapy until after delivery. Two months after delivery, the patient underwent total thyroidectomy to permit evaluation for metastatic disease and monitoring for recurrence by Tg levels. The thyroid pathology was benign.

**Fig. 2:**
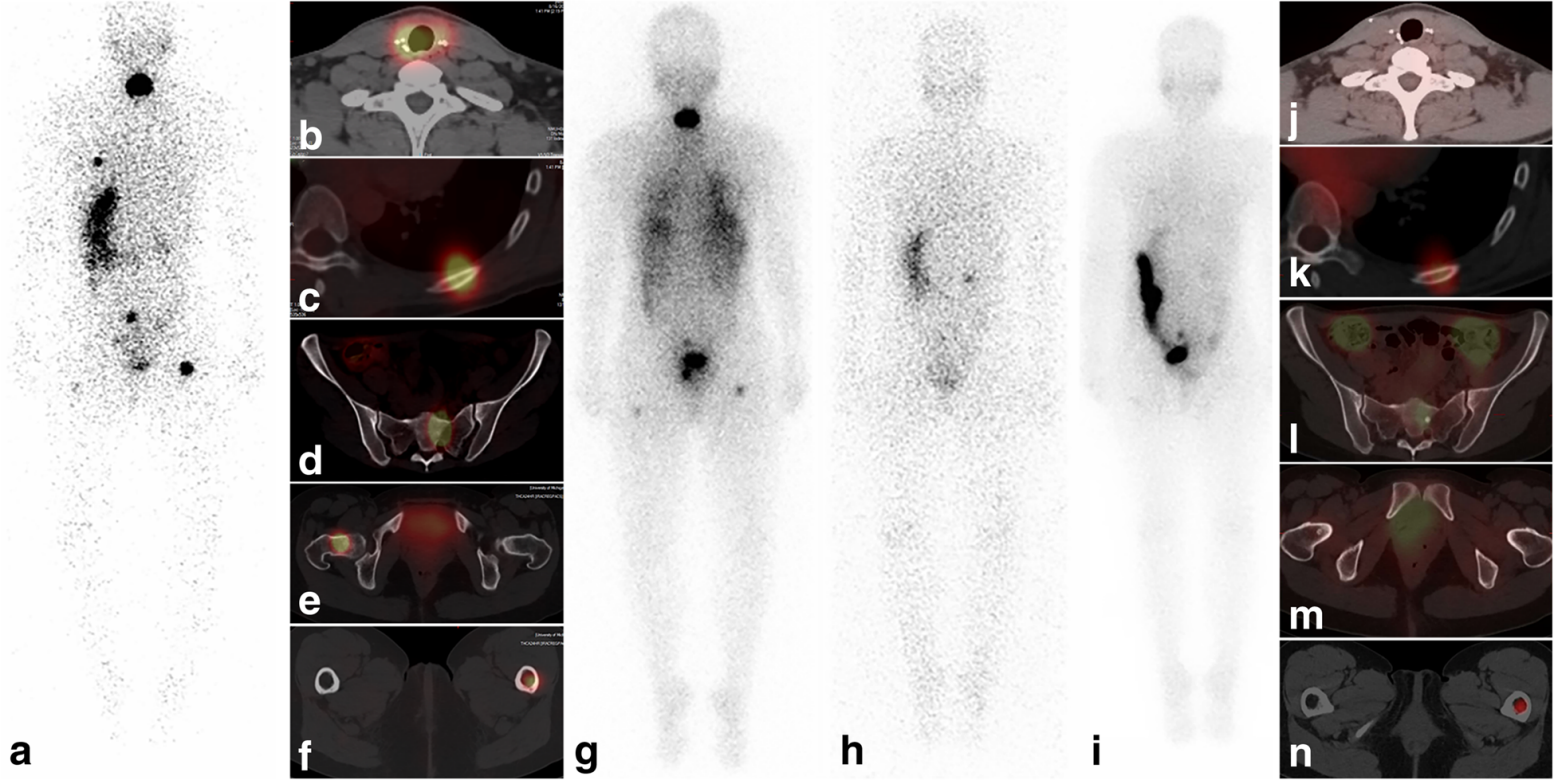
Nuclear medicine imaging at initial evaluation and subsequent follow-up of metastatic struma ovarii. Diagnostic 131-I planar scan, posterior view (**a**) demonstrates central neck activity and abnormal activity in the left hemithorax, central pelvis and bilateral proximal femurs. Diagnostic SPECT/CT demonstrates thyroid remnant tissue in the thyroidectomy bed (**b**) and skeletal metastatic disease localized to left 9th rib (**c**), sacrum (**d**) and bilateral proximal femurs (**e**, **f**). Post-therapy 131-I planar scan, posterior view (**g**) demonstrates diffuse lung activity consistent with miliary pulmonary metastases in addition to focal central neck activity and skeletal metastatic foci. Follow-up diagnostic planar 131-I scan, posterior view (**h**) demonstrates physiologic radiotracer activity in the stomach, and urinary activity in the right renal collecting system and bladder, without foci of abnormal activity. Post-therapy 131-I scan, posterior view (**i**) obtained after 100 mCi therapeutic challenge ascertained resolution of pulmonary miliary metastatic disease, but residual foci of activity were demonstrated in the left hemithorax and pelvis and confirmed on SPECT/CT in the left ninth rib (**k**), sacrum (**l**), and left proximal femur (**n**) consistent with a partial therapeutic response in the skeleton. Focal sclerosis without focal 131-I uptake is demonstrated in the right femoral neck, consistent with treatment response and bone healing (**m**). Complete therapeutic response is demonstrated in the thyroidectomy bed (**j**)

Several months after delivery and cessation of lactation, the patient underwent L-T4 withdrawal and her Tg unexpectedly rose from 0.8 ng/mL (TSH 5.8 mU/L) to 113.7 ng/mL (TSH 46 mU/L). A concurrent diagnostic I-131 scan (1 mCi) with planar and SPECT/CT imaging revealed focal central neck activity consistent with thyroid remnant and the presence of iodine-avid skeletal metastatic disease involving the left ninth rib, bilateral proximal femurs, and sacrum (Fig. 2A-F). Following 320 mCi I-131 therapy based on blood dosimetry calculations, post-therapy scan revealed the additional finding of miliary pulmonary metastases (Fig. 2G). Due to high-quality SPECT/CT images, separate dedicated CT and bone scan were not necessary. She was subsequently treated with TSH suppression resulting in an undetectable Tg < 0.1 ng/mL (TSH 0.02 mU/L). In order to assess the interval response to I-131 treatment, follow-up evaluation after L-T4 withdrawal protocol was obtained nine months later, demonstrating a stimulated Tg of 0.2 ng/mL (TSH 36.4 mU/L) and negative diagnostic I-131 scan without focal abnormal radioiodine uptake in the neck or skeleton. As she was planning for a second pregnancy and her pulmonary disease was not visible on the prior diagnostic I-131 scan but became apparent only on the post-therapy scan, a 100 mCi I-131 therapeutic challenge was performed to eliminate any microscopic residual disease and more accurately ascertain the treatment response. The post-therapy I-131 scan demonstrated complete resolution of pulmonary metastatic disease; however, faint residual foci of activity were demonstrated in the left ninth rib, sacrum, and left proximal femur, consistent with a partial therapeutic response in the skeleton (Fig. 2I-N). Eight months later, Tg was < 0.1 ng/mL (TSH 0.02 mU/L). The patient continues to do well, being conservatively managed with active clinical and biochemical monitoring during L-T4 suppression therapy.

## Discussion

There are no established diagnostic and therapeutic approaches for the management of malignant struma ovarii. While some other authors advocate for total hysterectomy and bilateral salpingo-oophorectomy in the setting of metastatic disease [2, 8], only a unilateral salpingo-oophorectomy was performed in this case due to the patient’s desire for future fertility and unclear benefit of more extensive resection. A series of 53 patients demonstrated that independent of surgical approach, recurrence rates were only 7.5% at 25 years for malignant struma ovarii, although the patients did not have known metastatic disease at diagnosis, follow-up was often markedly less than 25 years, and staging and management strategies were variable [2]. The authors recommended a risk stratification approach in which patients with gross extra-ovarian extension, distant metastases at diagnosis, or concurrent primary thyroid gland cancer receive total thyroidectomy and radioactive iodine while others receive pelvic surgery alone. Potential concerns with this approach include the limited ability to identify distant metastases without thyroidectomy and I-131 scan, difficulty monitoring for local recurrence in the pelvis (as opposed to the neck for primary thyroid gland cancer), and limited data regarding long-term follow-up and risk of recurrence.

Total thyroidectomy in this patient permitted diagnostic scintigraphic evaluation which identified distant metastatic disease to the skeleton and guided I-131 treatment based on blood dosimetry calculations. The decision to perform thyroidectomy and aggressively evaluate for metastatic disease was made given the lack of high quality data on the risk for metastases in this rare condition and the difficulty monitoring for local recurrence or residual disease in the pelvis. Additionally, the presence of multiple mitotic figures on histopathology review indicated rapid tumor growth, which may be associated with decreased survival in thyroid cancer arising from the thyroid gland [9, 10]. The association between mitotic figures and increased risk has not been shown with struma ovarii, although studies are limited by small sample size with the largest study (n = 86) showing ≥1 mitosis per ten high powered fields (HPF) in 42% of biologically benign struma ovarii vs. 57% of malignant struma ovarii (p = 0.22) [4]. Furthermore, studies have shown that the histologic features that increase the risk of malignant behavior in this class of tumors include size of the struma ovarii greater than 5 cm and the presence of greater than 50% of the teratoma containing proliferating thyroid tissue, both of which were present in this case [11].

While the preablation stimulated Tg of 113.7 ng/mL is quite elevated and consistent with distant metastatic disease based on proposed cutoff of 47.1 ng/mL [12], it may be lower than expected given the extent of metastatic disease involving the lungs and bone, as other studies have found that Tg > 215 ng/mL is more likely with this extent of disease [13]. Additionally, the second preablation stimulated Tg of 0.2 ng/mL with bony metastases may be lower than expected. These findings raise the possibility that this tumor has reduced efficiency for producing Tg which can be seen in de-differentiated thyroid cancers [14]. However, the focal I-131 uptake within the metastatic lesions demonstrated on scans argues that the tumor remains differentiated, maintaining its capacity to concentrate therapeutic I-131. Thyroid cancers have demonstrated variability in Tg production, and therefore correlation of stimulated Tg levels with scintigraphy findings has been used for guiding therapeutic I-131 administration [15].

In patients with cancer in the thyroid gland, SPECT/CT imaging has been shown to be helpful in providing anatomic correlate for planar scintigraphy findings and accurate localization of metastatic disease [16], as was true in this case. An important caveat with the use of *diagnostic* I-131 scintigraphy is the risk of false reassurance due to the low I-131 activity (1–2 mCi) used for diagnostic purposes. The post-therapy scan can provide additional information regarding the presence of metastases and response to therapy [17]. In this case, the pulmonary metastases were initially not evident on diagnostic whole body I-131 scan but became apparent on the post-therapy scan. Subsequent follow-up evaluation with a diagnostic I-131 scan was negative for disease persistence, and the stimulated Tg was only 0.2 ng/ml; however, the second post-therapy scan showed only partial therapeutic response in the skeleton. This highlights the importance of integrating all clinical, histopathology, laboratory and diagnostic and post-therapy imaging information for clinical decision making in the management of advanced and metastatic thyroid cancer.

An interesting aspect of this case is the clinical presentation of malignant struma ovarii during pregnancy and the patient’s desire for further fertility. Estrogen is known to be a stimulator of benign and malignant thyroid cells in vitro [18]. As has been shown in breast cancer, estrogen may impact adhesion, invasion, migration and angiogenesis in thyroid cancer and it is known that estrogen and progesterone receptors are present in papillary thyroid cancer cells [18, 19]. Recent pregnancy may be associated with a transient increased risk of thyroid cancer although data are limited [20, 21]. hCG is known to stimulate TSH receptors and may also promote tumor growth during pregnancy [22]. In this case, it is possible that the patient’s pregnancy contributed to the increased size of the tumor, and thus the discovery was made during pregnancy rather than prior to conception. The patient was counseled regarding the possible increased risk during subsequent pregnancies, although her continued undetectable Tg under L-T4 suppression is encouraging that she will do well. No further imaging has been performed to date since a conservative management strategy has been chosen based on the patient’s and treating endocrinologist’s preference. Although her tumor may inefficiently produce Tg, the very low stimulated Tg < 0.2 ng/mL (TSH 36.4 mU/L) obtained prior to the second therapeutic I-131 administration and the currently undetectable TSH-suppressed Tg are reassuring. Considering the risks for equivocal or indeterminate results with PET/CT imaging at very low Tg levels or nonspecific findings on bone scan, and given the unclear benefit of further aggressive treatment in the current clinical and biochemical context, the plan is for conservative management with clinical and biochemical monitoring with serial Tg testing during L-T4 suppression; further I-131 therapy remains possible if justified by biochemical evidence of recurrence and/or progression during long-term clinical surveillance.

## Conclusions

This unusual case presents important considerations in the care of patients with malignant struma ovarii. First, staging with a diagnostic whole body radioiodine scan and stimulated Tg level following thyroidectomy needs to be considered in all patients with intermediate and high-risk histopathology findings. Second, the I-131 scan should include SPECT/CT imaging, which can demonstrate additional areas of metastases missed on planar imaging, allows for accurate anatomic correlation of metastatic lesions, and can impact treatment decisions [16]. Third, when I-131 treatment is given, a post-therapy I-131 scan should always be obtained two-seven days after treatment to assess for additional findings as compared with the diagnostic I-131 scan [17]. Finally, the role of sex hormones and hCG in thyroid cancer arising from both struma ovarii and the thyroid gland should be explored further.

## Abbreviations

hCG: Human chorionic gonadotropin
I-131: Sodium iodide-131
L-T4: Levothyroxine
SPECT/CT: Single photon emission computed tomography/Computed tomography
Tg: Thyroglobulin
TSH: Thyroid stimulating hormone
TTF-1: Thyroid transcription factor
